# Assessing the impact of China’s river chief system on enterprise pollution discharge

**DOI:** 10.3389/fpubh.2023.1268473

**Published:** 2023-12-15

**Authors:** Jianxiao Du, Bo Li

**Affiliations:** ^1^School of Accountancy, Shandong University of Finance and Economics, Jinan, China; ^2^School of Management, Tianjin University of Technology, Tianjin, China

**Keywords:** environmental policy, river chief system, enterprise pollution discharge, river governance, public health

## Abstract

The River Chief System (RCS), a pivotal environmental governance policy promoted by the Chinese government, has far-reaching implications for public health. This study aims to comprehensively assess the impact of RCS on corporate pollution emissions, emphasizing its role in improving environmental quality and safeguarding public health. Utilizing a database of industrial enterprises and data from heavily polluting enterprises spanning 2003 to 2013. Manual collation of RCS policy implementation across prefecture-level cities during the same period. Application of the DID method to verify the impact of RCS on the extent of corporate pollution emissions. RCS significantly reduces pollution emissions from enterprises. Heterogeneity analysis reveals RCS to be more effective in addressing visible pollutants in rivers, non-provincial capitals, and heavily polluting industries, resulting in a notable reduction in pollution emissions. Mechanism testing underscores the importance of increasing government attention to environmental protection and strengthening environmental regulation as key factors contributing to RCS’s success in reducing pollution emissions from enterprises. Additionally, the study finds that improving the business environment of enterprises, measured through the marketization index, enhances the effectiveness of RCS in improving river pollution by enterprises. This study introduces a new perspective on examining the pollution reduction and abatement effects of RCS, addressing a gap in micro-level research. The findings not only contribute to the understanding of RCS’s impact on pollution but also offer valuable insights for governments and policymakers in promoting the further development and implementation of RCS policies. The results of this research are of significant importance in strengthening environmental governance and safeguarding public health. By effectively controlling corporate pollution emissions, RCS contributes positively to improving environmental quality and, consequently, enhancing public health outcomes.

## 1 Introduction

In recent years, the world has faced an urgent environmental challenge with escalating river pollution due to rapid industrialization and urbanization. Rivers, as vital water resources and ecosystem carriers, hold immense significance in supporting human life, industrial production, and ecological balance (1, 2). However, the frequent occurrence of pollution incidents in China’s river basins, caused by extensive wastewater and industrial pollutant discharges, as well as illegal dumping (3, 4), poses a grave threat to both human life and the ecological environment (5, 6). This issue directly impacts public health, as contaminated water sources can trigger outbreaks of water-related diseases and have long-term repercussions on ecosystems and food chains, thus affecting people’s well-being.

To combat the cyanobacteria crisis in Lake Taihu, an innovative watershed governance model known as the RCS was first introduced in Wuxi City, Jiangsu Province, China in 2007 (7). Unlike many other Chinese environmental policies dictated by the central government, the inception and propagation of the RCS represent a grassroots institutional enhancement that redefines the responsibilities for pollution control (8). The RCS is a locally driven initiative for managing water environments within river basins, with the primary goal of bolstering river pollution control and ecological preservation by clarifying the roles and oversight of local governments (9, 10). Under this policy, local authorities are mandated to appoint officials specializing in river management, referred to as river chiefs, who bear the responsibility for safeguarding, overseeing, and maintaining river basins (11). Subsequently, other regions have initiated trial implementations of the RCS (12). As of June 2018, this system has been universally adopted across the nation and has yielded significant improvements in in river basin pollution management (13). The introduction of the RCS has, to a certain extent, amplified the sense of responsibility and operational efficiency of local governments, providing robust support for the preservation of river ecosystems. The enhanced participation and governance of local governments have resulted in improved water quality, a reduction in pollution sources, and a more favorable ecological balance. These developments have had a positive impact on public health by lowering the health risks associated with pollution.

In river pollution management, enterprises play a critical role as the primary driving force behind economic development, and their significance should not be underestimated (14). The production activities of enterprises often involve substantial wastewater and pollutant discharge, as well as resource utilization. If not managed properly or lacking environmental awareness, these activities can cause irreparable damage to river water quality and the ecological environment. Regrettably, previous studies have mainly focused on data analysis at the provincial and prefectural levels (15), with less attention given to the actual performance and impacts of enterprises on river pollution. Additionally, there has been a lack of systematic research on how the promotion of the RCS affects enterprise behavior at the prefectural level. Understanding the actual performance and impacts of enterprises in river pollution is crucial for public health, as it enables the identification of pollution sources and the implementation of effective measures to reduce the risk of public exposure to pollutants.

Simultaneously, flat management is emerging as a distinctive approach to water pollution control. This model accentuates the active involvement and accountability of businesses with the goal of curbing pollutant discharge and enhancing the overall water quality. It underscores the active participation of businesses in water resource management and conservation, prioritizing collaboration and transparency in mitigating pollution while advancing sustainable development.

Although at first glance, flat management and the river chief system may appear to center on different aspects, they are intricately interconnected. Companies assume a pivotal role in water pollution management, with the flat management approach underscoring the significance of active corporate engagement, while the river chief system governs corporate conduct through regulations and the delineation of responsibilities. The combination of these two approaches is vital in comprehending the implications of corporate pollution discharges and their impact on public health.

In contrast to the previous practice of segmenting pollution management along administrative boundaries, the RCS streamlines the managerial hierarchy by instating river chiefs, thereby clarifying the responsibilities and assignments of each accountable party. This approach places a greater emphasis on participation, collaboration, and the establishment of long-term mechanisms, bolstering the transparency of governance responsibilities and supervision mechanisms. To a certain extent, a flattened management framework has been established for the oversight of specific rivers. The implementation of this flattened model has played a pivotal role in enhancing water environmental governance and advancing the sustainable utilization use and preservation of water resources.

This dissertation aims to investigate the relationship between the RCS and corporate pollution discharges. By synthesizing and analyzing relevant literature and utilizing data on industrial and polluting enterprises in China from 2003 to 2013, a progressive double difference (stagger-DID) model is employed to examine the impact of RCS on corporate river pollution and its underlying mechanisms. We selected the timeframe from 2003 to 2013 for our study due to a series of significant events and policy shifts closely tied to environmental governance during this period. This decade marked several pivotal moments, including the initiation and refinement of the “river management system.” Furthermore, national policies such as the Eleventh Five-Year Plan (2006–2010) and the Twelfth Five-Year Plan (2011–2015) underscored the Chinese government’s dedication to environmental sustainability. These plans implemented targeted measures aimed at curbing pollution, enhancing ecological protection, and fostering sustainable development. Additionally, this time frame aligns with heightened global attention to environmental issues, reflecting an era where environmental protection held a prominent position on the international agenda. The increased regulation of environmental governance during this period enriches our analysis, allowing us to investigate the impact of the “river chief system” in addressing pollution and promoting sustainable environmental practices in China within a broader global context.

In summary, the period spanning 2003 to 2013 presents a diverse landscape of environmental initiatives, policy adjustments, and global awareness that underscores the significance and validity of our study. The convergence of these elements renders this time period crucial for evaluating the effectiveness of the “river chief system” in mitigating pollution and advancing sustainable environmental practices in China.

This study contributes significantly in the following three areas. Firstly, by focusing on the micro-enterprise level, we systematically investigate the implementation effects of RCS. This research offers empirical evidence at the micro-level, bridging the existing gap in research data and providing a more comprehensive perspective for in-depth discussions regarding the economic impacts of the river management system on enterprises. Secondly, through heterogeneity analysis, we unveil the limitations of the river management system, particularly in managing deep, unobservable pollutants. Simultaneously, we clarify the substantial impact on reducing pollution emissions from enterprises in non-provincial capital areas and heavily polluted industries. This insight provides a scientific basis for the implementation of targeted environmental protection measures by both governments and enterprises. Finally, we delve into the mechanisms behind these effects, with a specific focus on two crucial dimensions: governance capacity and governance willingness. We explore topics ranging from enhanced environmental regulation and government concern for environmental protection to the level of marketization. This deeper understanding equips the government with more targeted decision support to promote the green innovation transformation of enterprises. Our findings not only guide governments in formulating more effective environmental policies and measures to support green innovation but also offer firms clearer environmental objectives and action plans. Furthermore, while pollution is typically addressed internationally through taxation or institutional means, China, as the world’s largest developing country, has, for the first time, adopted a reciprocity-based approach of responsibility and obligation to address environmental concerns. This experience and approach provide valuable lessons and inspiration for other emerging economies to replicate similar environmental governance policies, thereby establishing the “China model” of pollution control in developing countries and offering crucial support for achieving the sustainable development goals.

## 2 Literature review and research hypotheses

### 2.1 Literature review

RCS, as a significant flat environmental governance mechanism, has achieved remarkable progress in enhancing river water quality and strengthening the government’s pollution control capabilities compared to the conventional approach of controlling pollution by administrative units. Consequently, it has gained substantial attention from domestic and international scholars (16–19). With the increasing prominence of global environmental issues, improving river water quality has become a crucial objective in safeguarding both the ecological environment and human health. Hence, conducting an in-depth study on the effectiveness of RCS holds immense significance.

Extensive research on this hot topic has been conducted by foreign scholars. These countries often adopt governance approaches based on administrative units, implementing a series of measures to address water pollution. For instance, in the United States, comprehensive legal regulations are enforced to strictly manage pollution emissions, accompanied by significant fines for violations, leading to gradual improvements in water pollution (20). Germany utilizes inconsistent and low-diversity governance methods to tackle pollution caused by agricultural nitrogen surplus, achieving sustainable environmental development (21). The United Kingdom government controls pollutant emissions through strict legislation, requiring industrial wastewater to meet discharge standards, prohibiting unauthorized sewage discharge, and investing in the construction of sewage treatment plants and related infrastructure (22). France employs interception and treatment measures by relocating factories, continuously amending and improving legal systems, and mobilizing funds from multiple sources for river management, resulting in improved environmental conditions (23). These countries have implemented governance measures tailored to different situations and issues, combining the formulation and enforcement of laws and regulations, thereby achieving certain effectiveness.

In previous studies examining the effectiveness of RCS, scholars have derived valuable insights through theoretical and empirical analysis. For instance, Li et al. conducted an evaluation of RCS implementation in China and observed that it led to improvements in river water quality to a certain extent, effectively controlling pollution emissions from enterprises. Similarly, Gao et al. evaluated the practical application of RCS and found substantial results in reducing pollution discharges by enterprises. Wang and Xiong’s (24) study further corroborated these findings. Collectively, these studies provide empirical evidence supporting the effectiveness of RCS policies.

However, it should be noted that RCS still has some limitations in terms of enterprise pollution emissions. Some studies have pointed out that although the implementation of RCS has a certain inhibiting effect on the pollution emission of some enterprises, there are still some enterprises that evade regulation and discharge illegally (25). Moreover, Feng and Liao (26) emphasized the need for further reinforcement of RCS in terms of regulatory enforcement and penalties to effectively achieve the objective of managing enterprise pollution emissions. These findings remind us that RCS policies need to focus on the strength of regulation and enforcement, as well as the enhancement of penalties for violating enterprises in the implementation process.

Apart from its impact on controlling pollution emissions, RCS has been analyzed from other perspectives regarding its influence on enterprises (27, 28). On one hand, the implementation of RCS subjects enterprises to more stringent environmental regulations, prompting them to increase investment in environmental protection and adopt cleaner and sustainable production methods (29). This heightened environmental regulation can drive enterprises to enhance environmental management and engage in technological innovation, thereby promoting green development (30). On the other hand, the implementation of RCS also presents development opportunities for enterprises by guiding them toward technological innovation and green transformation, ultimately improving their environmental competitiveness and sustainability (31). This suggests that RCS provides positive incentives for enterprises to a certain extent, encouraging them to take positive actions in environmental protection (8).

To summarize, the relationship between RCS and enterprise pollution emissions is a complex and significant research area. Existing studies indicate that RCS has a certain level of control over enterprise pollution emissions. However, it also encounters challenges and limitations that need to be addressed.

### 2.2 Research hypotheses

Firstly, compared to the traditional approach of pollution control by administrative units, RCS has strengthened requirements in terms of the supervision mechanism. It introduces a mechanism of social supervision and public participation, demanding that enterprises comply with environmental protection regulations and restrictions (30). This will compel enterprises to enhance their control and management of pollution discharges, ensuring that their emissions fall within the scope of regulatory requirements and minimizing the adverse impact on the river ecosystem. Secondly, the implementation of RCS contributes to an increased awareness of environmental responsibility among enterprises (32). Enterprises will attach greater importance to environmental protection and recognize the significance of pollution emissions for their reputation and sustainable development. Under public and regulatory scrutiny, enterprises may voluntarily take measures to reduce pollution emissions, avoiding exposure and penalties.

Furthermore, to meet the requirements of RCS, enterprises may increase their investment in research and development (R&D) and promote technological innovations and improvements to reduce pollution emissions (31). This may involve the adoption of more efficient pollution control equipment, the implementation of cleaner production technologies, and the optimization of production processes. As technology advances, enterprises are expected to lower their emission levels and achieve sustainable development (33). Moreover, the implementation of RCS encourages collaboration and coordination among the government, enterprises, and various sectors of society (25). By establishing communication mechanisms and platforms for cooperation across different sectors, enterprises can work together with the government, environmental organizations, and other stakeholders to establish emission reduction targets and measures, as well as share resources and experiences. Such coordination and cooperation can enhance the effectiveness of emission reduction efforts and encourage enterprises to actively fulfill their environmental responsibilities.

In conclusion, the implementation of RCS results in a decrease in pollution emissions through the enforcement of mechanisms, the enhancement of environmental responsibility awareness, the promotion of technological innovation and improvement, and the facilitation of collaboration and coordination. Based on these findings, we put forth Hypothesis 1.

*H1*: RCS has a negative impact on enterprise pollution emissions.

First of all, it is different from the previous mode of pollution control by administrative units, in which water environment management was the responsibility of the environmental protection departments at all levels of government, and the management method was usually based on administrative orders and regulatory means. In contrast, RCS constructs a flat approach, forming a more flexible and collaborative management system, with responsibilities assumed by river chief organizations, including river chiefs and river chiefs’ offices, forming a multi-party participatory mechanism involving the government, social organizations, and the public (34).

Second, the government’s attention to environmental protection is one of the prerequisites for the effective implementation of RCS (35). RCS, as an institutional arrangement for river management and protection, requires governmental departments to attach great importance to environmental protection and provide the necessary support and resources for it (34). Simultaneously, the government can establish more stringent emission standards and access criteria based on the needs of river ecosystems and water resource conservation requirements. This compels enterprises to adopt more effective pollution control measures and reduce pollutant emissions to meet the government’s stipulated requirements. The government’s attention can lead to stricter enforcement of environmental protection policies, which will have a positive effect on the pollution emissions of enterprises (36).

In addition, the government’s attention to environmental protection can influence public opinion and the market behavior of society, which will have a positive effect on the pollution emission of enterprises (37). By strengthening environmental publicity and education, the government increases public awareness and participation in environmental protection, forming preference and support for environmentally friendly enterprises. At the same time, the government’s attention to environmental protection will also cause the market to pay attention to environmentally friendly enterprises (38), for example, investors and consumers are more inclined to support and choose environmentally friendly enterprises. This change in market demand will prompt enterprises to consciously reduce pollution emissions to meet market demand, thus affecting their pollution emission behavior.

To summarize, RCS reduces the pollution emissions of enterprises by increasing the government’s attention to environmental protection. The government’s attention guides enterprises to consciously reduce pollution emissions and realize the goal of sustainable development by setting strict standards, guiding market demand, and supporting technological innovation. Based on this, we propose hypothesis 2.

*H2*: RCS reduces enterprise pollution emissions by increasing government attention to environmental protection efforts.

Firstly, unlike the previous short-term measures taken by administrative units, RCS introduces a long-term mechanism that emphasizes continuous improvement and long-term governance and is no longer a temporary administrative act (25). By establishing a robust assessment and evaluation mechanism, the performance of river chiefs and related entities is evaluated, incentivizing and constraining all stakeholders to participate in water environmental protection collectively. Secondly, the implementation of RCS strengthens the environmental access audit and management of enterprises (31). Enterprises are required to meet more stringent environmental protection requirements to obtain business licenses or project approvals. This prompts them to consider environmental protection factors during project planning and operation and implement pollution prevention and control measures accordingly to meet the access requirements.

At the same time, to comply with RCS requirements, enterprises may be motivated to upgrade their technologies and adopt more advanced pollution control equipment (39). The implementation of RCS can incentivize enterprises to invest in research and development and embrace environmentally friendly production processes and technologies to reduce pollutant emissions (40). Additionally, the government can provide appropriate policy support and financial incentives to encourage enterprises to adopt cleaner production technologies and pollution control measures (41). The implementation of RCS can also enhance the disclosure and transparency of environmental information to enterprises (42). By publicizing pollution emission data and environmental conditions of enterprises, as well as strengthening public opinion monitoring, the public, and the media can supervise and evaluate enterprises’ environmental performance (43). This will push enterprises to strengthen their environmental management and respond to public and market pressures to reduce pollution emissions.

In summary, the RCS reduces enterprise pollution emissions through enhanced environmental regulation. The government’s supervision and enforcement, environmental access audit and management, technology upgrades, and introduction of pollution control equipment, as well as the improvement of environmental information disclosure and transparency, collectively drive enterprises to reduce pollution emissions and achieve environmental protection goals. Based on these observations, we propose Hypothesis 3.

*H3*: RCS reduces enterprise pollution emissions by strengthening environmental regulation.

First, the business environment can provide economic incentives for enterprises and guide them to reduce pollution emissions through economic means (44). In the implementation of RCS, the government can quantify and economically manage the pollution emissions of enterprises through market-based means, such as carbon emissions trading and emissions licensing system. By quantifying and trading pollution emissions and introducing market mechanisms, enterprises will face economic cost considerations (45). High-emission enterprises will be subject to higher cost pressure, while low-emission enterprises can gain economic benefits by reducing emissions. This economic incentive mechanism will motivate enterprises to take proactive measures to reduce pollution emissions to reduce economic costs or gain additional economic benefits.

Second, we choose to introduce the marketability index as a proxy indicator of the business environment of enterprises, reflecting the measures taken and the performance of enterprises in environmental protection and sustainable management. The marketability index can improve the transparency of enterprises’ environmental information and public monitoring (46). By disclosing enterprises’ pollution emission data and environmental status, as well as strengthening public opinion monitoring and environmental assessment, the MMI can provide the public and the media with information about the environmental performance of enterprises (47). Public and market recognition of environmentally friendly enterprises will increase, while pressure and condemnation of high-pollution-emitting enterprises will also increase. Enterprises will be more inclined to reduce pollution emissions to maintain their reputation and market competitiveness to satisfy consumers’ and investors’ demand for environmental protection.

Furthermore, the Marketization Index can also facilitate the growth of the environmental protection industry and spur technological innovation (48). As environmental awareness improves and market demand increases, the environmental protection industry will have access to more opportunities and market potential (49). Enterprises will actively invest in research and development, as well as adopt environmental protection technologies, to enhance productivity and mitigate pollution emissions. The presence of the Marketization Index will generate additional investment and development prospects for the environmental protection industry, promoting technological innovation and facilitating green development.

In summary, enterprises’ business environment (market-based index) plays a positive regulatory role in RCS in reducing enterprises’ pollution emissions, and through economic incentives, information transparency, and market competition, it can guide enterprises to reduce pollution emissions. Based on this, we propose hypothesis 4.

*H4*: enterprises’ business environment (market-based index) plays a positive regulatory role in RCS in reducing enterprises’ pollution emissions.

## 3 Model specification and variable description

### 3.1 Model specification

Drawing on the existing literature (38–42), we propose a generalized DID (Difference-in-Differences) model with a panel fixed effects model for baseline estimation. The baseline model is set up as follows:


$$\text{lox}\_w_{i,t}=\beta_{0}+\beta_{1}{\text{policy}}_{i,t}+\beta_{i}{\text{controls}}_{i,t}+\gamma_{i}+\delta_{t}+\varepsilon_{i,t}$$

Where $\text{lox}\_w_{i,t}$ denotes the degree of contamination, $\beta_{0}$ denotes the intercept term, and $\text{polic}y_{i,t}$ denotes a dummy variable for whether or not RCS is implemented. The i and t represent prefecture-level city and time, respectively. $\gamma_{i}$ denotes individual fixed effects, controlling for influences that vary across individuals. $\delta_{t}$ denotes time-fixed effects, controlling for influences that vary over time. The residual term is denoted by $\varepsilon_{i,t}$.

### 3.2 Data sources and variable description

#### 3.2.1 Data source and sample handling

In this study, data from industrial enterprise spanning the years 2003 to 2013 were employed. Additionally, we manually curated historical RCS implementation data for each prefecture-level city, which served as the study sample. The GDP data and population data of each prefecture-level city, in constituting the control variables, were obtained from the CSMAR database. he study employed the DID method to assess the impact of RCS on the extent of pollution emissions by these enterprises.

#### 3.2.2 Variable description

(1) Dependent Variable: River Pollution Level (low). Based on the First National Pollution Census Report, this study selects major industries such as Chemical Oxygen Demand (COD), Ammonia Nitrogen (NH3), Volatile Phenols, and Petroleum as indicators of water pollution industries. Additionally, following the data processing method used by Xu et al. (50), this study uses Chemical Oxygen Demand (COD) to measure the pollution level of enterprises on rivers. The COD data is obtained from the Chinese Industrial Enterprise Database and the Chinese Industrial Enterprise Pollution Database from 1998 to 2014, and it is merged with the pollution emission repository by matching industrial enterprise names, years, and pollution emissions.

(2) Independent Variable: Implementation of RCS. This study identifies the implementation year of RCS in different regions by searching and collecting official documents released and implemented by local municipal government websites. The data on RCS implementation is manually compiled. Based on the starting year of RCS implementation, regions that implemented RCS and subsequent years are defined as 1, while regions that did not implement RCS or implemented it before the RCS implementation year are defined as 0. This data covers the implementation of RCS from 2008 to 2016.

Drawing on a range of literature (51–53) that examines the impact factors of RCS and enterprise water pollution, this paper selects the following control variables: enterprise fixed assets (lasset), enterprise profit (profit), enterprise employment (person), enterprise debt ratio (FR), and gearing ratio (ROC), and the reasons for selecting the above control variables are as follows:

##### 3.2.2.1 Enterprise total assets (lasset)

The enterprise’s total assets are an indicator of the overall financial size of the enterprise. By controlling for this variable, the potential impact of enterprise size on pollution emissions can be considered.

##### 3.2.2.2 Enterprise profit (profit)

the total profit of the enterprise reflects the business condition of the enterprise. By controlling this variable, it is possible to consider the potential impact of an enterprise’s economic situation on pollution emissions.

##### 3.2.2.3 Employment size (person)

the number of enterprise employees reflects the size of the enterprise and the use of the labor force. By controlling this variable, the potential impact of labor force size on pollution emissions can be considered.

##### 3.2.2.4 Debt-to-asset ratio (FR)

Enterprise Debt Ratio is the ratio of total liabilities to total assets, reflecting the financial risk and solvency of the enterprise. By controlling this variable, the potential impact of an enterprise’s financial status on pollution emissions can be considered.

##### 3.2.2.5 Return on assets (ROC)

The return on assets is the ratio of total profit to total assets, reflecting the capital structure and financial stability of the enterprise. By controlling this variable, the potential impact of an enterprise’s financial situation on pollution emissions can be considered.

By choosing the above control variables, the impact of water pollution flattening treatment on the pollution discharge of enterprises can be analyzed more accurately, while controlling other factors that may affect the relationship. The specific treatment method is shown in Table 1.

**Table 1:** Definitions and descriptions of key variables.

Type	Variable	Description	Data processing	Variable source
Dependent Variable	*lox_w*	The logarithm of chemical oxygen demand	Taking the logarithm of chemical oxygen demand and winsorize.	We matched the database of Chinese industrial enterprises from 2003 to 2013, and the pollution database of Chinese industrial enterprises with the names and years of the industrial enterprises and merged them together. Subsequently, we calculate the COD (chemical oxygen demand) content and take its logarithm as a measure of how polluted the river is.
Independent Variable	*policy*	Implementation of RCS	Manually compiling the implementation status of RCS in prefecture-level cities over the years.	We collected data on the implementation of the river management system by visiting the websites of municipal governments in each region and searching for official documents on the issuance and implementation of the river management system in each region as a way of confirming the starting year of the implementation of the system in each region, as well as by hand-collecting the data on the implementation of the system.
Control variable	*lasset*	Enterprise size	The logarithm of enterprise total assets	Csmar database
	*profit*	Enterprise profit	The logarithm of the total profit of the enterprise	
	*person*	Employment size	Number of employees in the enterprise	
	*FR*	Debt-to-asset ratio	Measured as the ratio of total liabilities to total assets	
	*ROC*	Return on assets	Measured as the ratio of total profit to total assets	

The descriptive statistics of the main variables of this study are detailed in Table 2. It can be seen from Table 2 that the mean value of Chemical Oxygen Demand (COD) is 1.813, the minimum value is 0, and the maximum value is 6.894. The standard deviation is large than the mean, which indicates that there is a certain degree of fluctuation in COD in different enterprises. This volatility may be related to factors such as geographic location and climate, which have significant variability. In terms of policy variables, the mean value of whether RCS is implemented or not is 0.148 and the median value is 0. This indicates that the data shows a right-skewed distribution. A right-skewed distribution indicates that there is a long tail on the right side of the data, i.e., there are some larger values that pull up the mean. This indicates a large variation in the effect of implementing the RCS policy between cities, with only some cities having a more pronounced effect. This may be because the implementation of RCS policies requires a high level of organizational management and financial investment, while factors such as policy implementation, regulatory efforts, and the willingness of enterprises to cooperate between different cities may also have an impact on the effectiveness of RCS. In contrast, some developed cities or capital cities have more resources and advantages and can form an efficient and complete administrative system, while some less developed or remote cities have difficulties in keeping up. The standard deviations of the control variables are small relative to the mean, indicating that there is little change in these variables, which may be related to factors such as industry characteristics.

**Table 2:** Descriptive statistics for the main variables.

Variable type	Symbol	Symbol size	Mean	Sd	Min	P50	Max
Dependent variable	*lox_w*	433,733	1.813	1.693	0	1.386	6.894
Independent variable	*policy*	516,660	0.148	0.355	0	0	15
Control variable	*lasset*	458,919	9.845	1.961	0	9.838	18.95
	*profit*	376,034	7.986	2.197	0	7.989	18.43
	*person*	509,996	5.507	1.162	0	5.509	12.32
	*FR*	461,828	0.443	0.192	−0.454	0.461	11.09
	*ROC*	461,620	0.080	0.174	−7.428	0.037	8.517

## 4 Empirical results and analysis

### 4.1 Baseline regression

After completing the relevant data preparation, this study used DID and panel fixed effects models to test Hypothesis 1. Table 3 reports the results of the regression of RCS on enterprises’ COD. In particular, column (1) shows the regression results of chemical oxygen demand, revealing the relationship between the initial RCS and enterprise COD. Subsequent columns (2) through (6) gradually incorporate control variables. In column (6), where fixed effects and control variables are added, the estimated coefficient is −0.059, which is significant at the 1% level of significance. This implies that the level of pollution emission is significantly lower in enterprises implementing RCS policies compared to those in areas where RCS policies are not implemented. Therefore, hypothesis H1, which states that there is a negative correlation between RCS and the degree of pollution emission by enterprises, is verified.

**Table 3:** Regression results of the COD and RCS.

	Fixed effects of panel data					
	(1)	(2)	(3)	(4)	(5)	(6)
	*lox_w*	*lox_w*	*lox_w*	*lox_w*	*lox_w*	*lox_w*
*Policy*	−0.055** (−2.57)	−0.056** (−2.56)	−0.056*** (−2.58)	−0.059*** (−2.69)	−0.059*** (2.75)	−0.059*** (−0.26)
*Lasset*		0.037***	0.033***	0.026***	0.026***	0.027***
		(12.74)	(11.60)	(8.85)	(8.85)	(9.14)
*Profit*			0.017***	0.014***	0.014***	0.011***
			(9.48)	(7.81)	(7.80)	(5.90)
*Person*				0.082***	0.082***	0.081***
				(12.46)	(12.46)	(12.30)
*FR*					−0.007	0.009
					(−0.31)	(0.40)
*ROC*						0.121***
						(5.21)
Year fixed effect	Yes	Yes	Yes	Yes	Yes	Yes
Firm fixed effect	Yes	Yes	Yes	Yes	Yes	Yes
Observations	401,834	359,605	359,605	359,605	359,605	359,605
Adj. R-squared	0.768	0.771	0.771	0.772	0.772	0.772

***, **, and * represent significance levels of 1, 5, and 10%, respectively.

In most studies, NH3N is considered a critical assessment indicator that, in conjunction with COD, determines the pollution level in rivers. Recognizing that relying solely on COD may not accurately portray the true extent of river pollution, we conducted dedicated regression analyzes to investigate the connection between NH3N and the river management system. The empirical findings revealed an estimated coefficient of −0.036, significant at the 5% level, after incorporating fixed effects and control variables. This suggests a significant adverse influence of the RCS on NH3N, further substantiating our hypothesis H1, which posits a negative correlation between the RCS and the level of pollution discharge from enterprises. This supplementary analysis bolsters our examination of the RCS policy, showcasing its broad and adverse impacts on effectively controlling multiple pollution indicators (Table 4).

**Table 4:** Regression results of the NH3N and RCS.

	Fixed effects of panel data
	***NH3N***
	***lox_w***
*Policy*	−0.036**
	(−2.29)
Year fixed effect	Yes
Firm fixed effect	Yes
Control	Yes
Observations	218,621
Adj. R-squared	0.68

***, **, and * represent significance levels of 1, 5, and 10%, respectively.

### 4.2 Robustness test

#### 4.2.1 Parallel trend test

**Figure 1:**
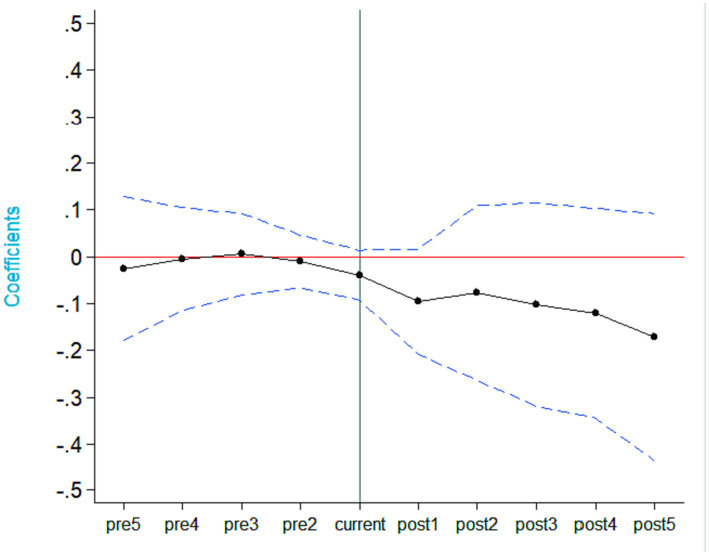
Parallel trend test results.

One of the key assumptions of the Difference-in-Differences (DID) method is that there are no significant differences between the treatment group and the control group before the occurrence of an external shock. This study draws on the method proposed by Xu et al. and utilizes the parallel trends test to validate this assumption. The specific steps are as follows:

First, the control group and treatment group are selected. Next, the difference between each year and the year of RCS implementation is calculated for the treatment group. These differences are used to generate binary variables for each year, indicating whether it is before or after RCS implementation for both the treatment and control groups. Finally, the generated binary variables are introduced into the regression model, and a parallel trends hypothesis test graph, as shown in Figure 1, is plotted. In Figure 1, the vertical axis represents the regression coefficients for each binary variable, while the horizontal axis represents the virtual variables before and after the RCS implementation year. For example, “pre1” represents the difference between the treatment and control groups 1 year before RCS implementation, “post1” represents the difference 1 year after RCS implementation, and so on.

From Figure 1, it is evident that there are no significant differences between the treatment and control groups before RCS implementation, which satisfies the requirements of the parallel trends hypothesis test.

#### 4.2.2 Placebo test

**Figure 2:**
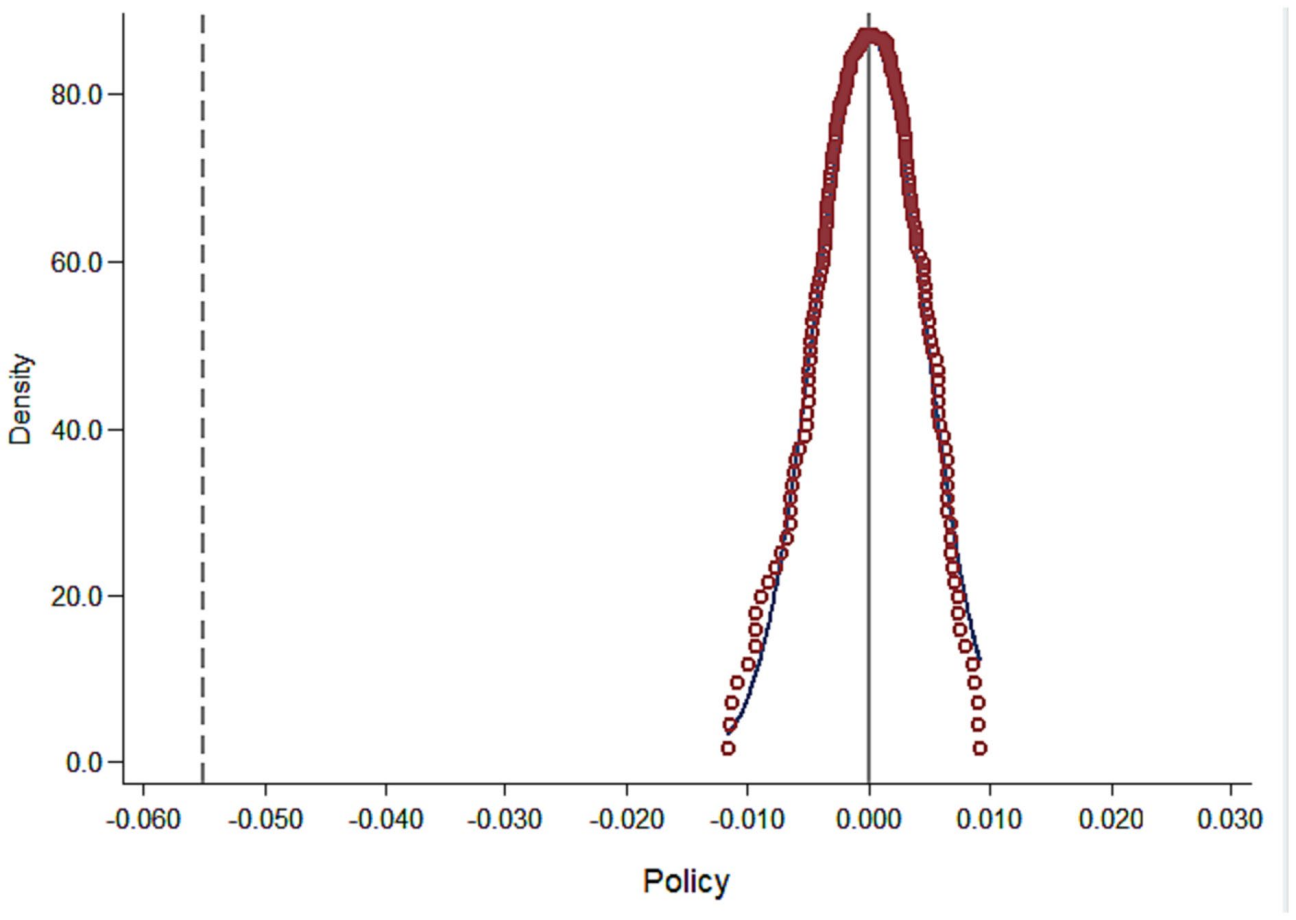
Result of the placebo test.

To ensure that the baseline regression results are not driven by random factors, this study refers to the research conducted by Gao et al. We randomly selected 22 cities from the entire sample of cities as the pseudo-experimental group (i.e., cities implementing RCS) for placebo testing. In each regression, we randomly selected 22 cities from the sample of 42 cities and assumed that these cities implemented RCS. The remaining cities were assumed to be the pseudo-control group and did not implement RCS. Since the selection was random, the pseudo-RCS dummy variables should not have a significant effect on the Chemical Oxygen Demand, and the estimated coefficients should be close to zero. To avoid interference from other rare events on the estimation results, we repeated the process 500 times. Figure 2 displays the distribution of p-values and the probability density of estimated coefficients for the 500 randomly selected treatment groups. The results show that the average value of the estimated coefficients is close to zero and is concentrated around zero in an approximately normal distribution. The true estimated coefficient of 0.01 is represented by the vertical line in Figure 2, located at the margin of the normal distribution. This indicates that the pseudo-RCS dummy variables do not have a significant effect, and thus the primary regression results of this study are not influenced by random factors.

To more accurately assess the impact of the RCS (a policy or measure) on river water quality, we conducted a comprehensive analysis that specifically excluded the potential interference of sulfur dioxide (SO_2_) emissions and soot emissions in the river on the results. We performed a baseline regression test to examine the relationship between the RCS and both SO_2_ emissions and soot emissions, aiming to determine if they had a significant effect on the outcomes of the study. Table 5 presents the results of this analysis, indicating that there is no significant correlation between RCS and sulfur dioxide emissions, as well as RCS and soot emissions. This suggests that the implementation of RCS had a significant negative impact on COD (Chemical Oxygen Demand) levels in the river, while no similar correlation was observed for the other pollutants. This finding carries important implications for our study.

**Table 5:** Heterogeneity of observed pollutants or unobserved pollutants.

	Fixed effects of panel data	
	Ammonia nitrogen	Industrial waste water
	*lox_w*	*lox_w*
*Policy*	−0.036**	−0.051
	(−2.29)	(−1.56)
Year fixed effect	Yes	Yes
Firm fixed effect	Yes	Yes
Control Variables	Yes	Yes
Observations	218,621	284,779
Adj. R-squared	0.68	0.75

Firstly, it demonstrates the effectiveness of the RCS policy in reducing COD levels. COD serves as a crucial water quality indicator, and elevated levels of COD can lead to significant adverse effects on river ecosystems and aquatic organisms. Therefore, the implementation of RCS can contribute to enhancing river water quality and protecting the ecological environment. Secondly, concerning sulfur dioxide emissions and soot emissions, these results indicate their relatively minor impacts on river water quality. Despite being prominent air pollutants, the absence of a significant association between them and RCS suggests the existence of other factors or environmental mechanisms that contribute significantly. These findings provide valuable insights for a more comprehensive comprehension of the environmental consequences of RCS. They suggest that increased attention should be directed toward COD control measures and further investigation of other factors that could influence river water quality. This approach will aid in the development of more effective strategies for water resource management, facilitating the protection and restoration of our valuable watershed ecosystems (Table 6).

**Table 6:** Sulfur dioxide emissions, smoke emissions, and RCS.

	Fixed effects of panel data	
	(1)	(2)
	*lso2pf*	*lsmokepf*
*Policy*	−0.026	−0.094
	(−1.35)	(−0.97)
Year fixed effect	Yes	Yes
Firm fixed effect	Yes	Yes
Control Variables	Yes	Yes
Observations	328,127	109,389
Adj. R-squared	0.83	0.80

### 4.3 Heterogeneity analysis

Heterogeneity analysis is crucial for studying the effects of RCS on pollution discharge from enterprises within cities, considering the variability that exists between enterprises at different administrative levels and pollution levels. This type of analysis enables us to comprehend the performance and disparities among enterprises operating at various administrative levels and pollution levels. For instance, factors like different industries, enterprise sizes, and technological capabilities can influence the effectiveness of water pollution control measures. By categorizing enterprises based on distinct characteristics and behaviors, and comparing the differences between these groups, we can gain a more precise understanding of the impact of RCS on enterprises. Consequently, this allows us to propose more accurate conclusions and recommendations. Heterogeneity analysis helps us uncover nuanced patterns, identify best practices, and tailor policy measures to specific enterprise types or sectors, ensuring that water pollution control efforts are targeted and effective.

#### 4.3.1 Heterogeneity of observed pollutants or unobserved pollutants

According to the findings presented in Table 5, significant differences were observed when analyzing the heterogeneity of pollutant observability about RCS and enterprise water pollution. The effectiveness of RCS implementation in reducing observable and unobservable pollutants varied significantly.

In this study, we selected ammonia nitrogen discharge in rivers as an indicator of observable pollutants. When the ammonia nitrogen content exceeds a certain threshold, it often leads to water body eutrophication, resulting in black and malodorous river water (54). Conversely, the discharge of industrial wastewater beyond the standard may contribute to elevated levels of heavy metals in the river, which are typically not directly observable (55). Therefore, for this study, we considered industrial wastewater discharge as an indicator of unobservable pollutants. We examined the heterogeneity between RCS and these two types of pollutants to determine if policy implementation had a significant impact on them. As shown in Table 5, we discovered a negative relationship between RCS and ammonia nitrogen discharge, indicating that the implementation of RCS had a significant negative effect on observable pollutants in the river. However, no significant correlation was observed between RCS and industrial wastewater discharge. This suggests that while RCS has achieved significant outcomes in controlling observable pollutants, similar associations were not found for unobservable pollutants.

Furthermore, we conducted additional analysis to examine the disparities in the effectiveness of RCS on the management of observable and unobservable pollutants, aiming to gain insights into the underlying reasons. Observable pollutants are primarily influenced by direct riverine pollutants, such as ammonia nitrogen discharged from agricultural and urban sources. The implementation of RCS can effectively reduce the concentration and impacts of observable pollutants by restricting the input of direct riverine pollutants through enhanced river management and monitoring. However, unobservable pollutants originate from more complex sources, including industrial wastewater and groundwater discharges, among others. These pollutants are typically found in a dissolved state and are not directly influenced by improvements in surface water quality. As a result, there is no apparent correlation between unobservable pollutants and the implementation of RCS. Addressing the challenge of unobservable pollutants requires a comprehensive approach that encompasses multiple areas, including industrial pollution control, water resource management, and environmental monitoring. This integrated approach is essential for reducing the discharge and impact of unobservable pollutants effectively.

In summary, RCS has achieved significant progress in controlling observable pollutants in rivers. However, managing unobservable pollutants poses ongoing challenges. To achieve comprehensive river management, it is crucial to strengthen collaboration with the industrial sector and related stakeholders. This collaboration should focus on developing a comprehensive management strategy that addresses both observable and unobservable pollutants. By doing so, we can safeguard the health of river ecosystems and ensure the sustainable utilization of water resources.

#### 4.3.2 Heterogeneity of administrative level

Heterogeneity analysis of provincial and non-provincial cities for the topic of RCS and enterprise water pollution, the results of the study Table 7 shows that there is a significant difference between provincial and non-provincial cities in the effectiveness of the implementation of RCS.

**Table 7:** Heterogeneity of administrative level.

	Fixed effects of panel data	
	Provincial cities	Non-provincial cities
	*lox_w*	*lox_w*
*Policy*	0.066	−0.077***
	(1.09)	(−3.29)
Year fixed effect	Yes	Yes
Firm fixed effect	Yes	Yes
Control variables	Yes	Yes
Observations	39,104	239,869
Adj. R-squared	0.81	0.77

Firstly, considering the geographical and economic structure, there are variations between provincial and non-provincial cities, which contribute to the divergent impacts of RCS implementation. Provincial cities are primarily characterized by a dominant tertiary industry, which has lower water resource demands and relatively lower levels of pollution emissions compared to the primary and secondary industries. This scenario makes it challenging for highly polluting enterprises to thrive (56). On the other hand, non-provincial cities have a higher proportion of primary and secondary industries, resulting in relatively higher levels of pollution emissions. Under identical conditions, the implementation of RCS demonstrates a more pronounced improvement in reducing pollution emissions in non-provincial cities.

Secondly, in terms of administrative and jurisdictional capacity, provincial cities possess advanced infrastructure, abundant resources, and well-developed transportation networks. They also have stricter and more comprehensive urban governance and management measures. Additionally, provincial cities tend to have a higher concentration of high-tech industries, a higher overall talent quality, stronger environmental awareness, and better execution capabilities among enterprises. Consequently, previous environmental protection policies have been better implemented and enforced in these cities, resulting in lower levels of pollution emissions. In contrast, non-provincial cities are predominantly characterized by traditional enterprises and heavy industries. They may have shortcomings in the comprehensive technical expertise of personnel, weaker environmental awareness, and more serious pollution emissions. After the implementation of RCS, more significant improvements have been observed in non-provincial cities.

In summary, significant differences exist between provincial and non-provincial cities in the promotion and implementation effects of RCS. This finding suggests the need to carefully consider the variations and characteristics of different geographical areas when formulating RCS. Corresponding governance measures should be tailored to local conditions to achieve more effective governance outcomes. Additionally, the government should increase awareness and publicity about RCS, promoting public consciousness of environmental protection. This will foster a collective effort throughout society to jointly advance resource governance and protection.

#### 4.3.3 Heterogeneity of pollution level

Heavily polluting industrial enterprises tend to exhibit high levels of COD emissions and a more severe degree of pollution in their surrounding areas. Conversely, enterprises in lightly polluting industries typically have lower COD emissions and a lighter degree of pollution. Consequently, the impact of RCS on pollution levels may vary between heavily polluted industries and lightly polluted industries. To account for this heterogeneity, this study refers to existing literature that classifies industries as either heavy or light polluters. By drawing on this literature, the 36 two-digit polluting industries examined in Table 5 were categorized into heavy and light polluting industries. The specific categorization of industries is presented in Table 8 (57), derived from the existing literature on the division between heavy and light-polluting industries.

**Table 8:** Classification of pollution levels of enterprises.

Categorization	Industry Code	Industry Name
Heavily polluting industrial enterprises	6、7、8、9、10、13、14、15、17、19、22、24、25、26、27、28、30、31、32、33、34、37、44、45、46	Coal Mining and Washing, Oil and Gas Mining, Ferrous Metal Mining and Processing, Nonferrous Metal Mining and Processing, Nonmetallic Mining and Processing, Agricultural and Food Processing, Food Manufacturing, Beverage Manufacturing, Textile Industry Leather, fur, feather (down) and its products industry, paper and paper products industry, arts, education and sporting goods manufacturing, petroleum processing, coking, and nuclear fuel processing industry, chemical materials and chemical products manufacturing, pharmaceutical manufacturing, chemical fiber manufacturing, plastic products industry, non-metallic mineral products industry, ferrous metal smelting, and rolling processing industry, non-ferrous metal smelting and rolling processing industry, metal products, transportation equipment manufacturing, electric power, heat production, and supply industry, gas production and supply industry, water production and supply industry Manufacturing, electric power, heat production, and supply industry, gas production and supply industry, water production and supply industry
Lightly polluting industrial enterprises	16、18、20、21、23、29、35、36、39、40、41	Tobacco products industry, textile and clothing, shoes and hats manufacturing, wood processing and wood, bamboo, rattan, palm and grass products industry, furniture manufacturing, printing and recording media reproduction, rubber products industry, general equipment manufacturing, special equipment manufacturing, electrical machinery and equipment manufacturing, communications equipment, computers and other electronic equipment manufacturing, instrumentation, and cultural, office machinery manufacturing

Following the grouped regressions, this study compares enterprises in heavily polluting industries with enterprises in lightly polluting industries, and the results are presented in Table 9. The findings reveal that RCS has a significant positive effect on reducing the degree of pollution emissions in heavily polluting industries, but not in lightly polluting industries.

**Table 9:** Heterogeneity of pollution level.

	Fixed effects of panel data	
	Lightly polluting industries	Heavily polluting industries
	*lox_w*	*lox_w*
*Policy*	−0.015	−0.070**
	(−0.50)	(−2.49)
Year fixed effect	Yes	Yes
Firm fixed effect	Yes	Yes
Control Variables	Yes	Yes
Observations	50,043	224,080
Adj. R-squared	0.717	0.775

After the implementation of RCS, the government has strengthened regulations for enterprises in heavily polluting industries and implemented stricter penalties for violations of environmental protection regulations. These enhanced regulations and penalties have encouraged enterprises to comply more closely with environmental protection measures, thereby reducing the extent of environmental pollution caused by these enterprises. Consequently, RCS demonstrates a significant positive effect on reducing pollution emissions among enterprises operating in heavily polluting industries. In contrast, enterprises in lightly polluting industries generally have lower pollution levels. The government may adopt a relatively flexible approach to enforcing environmental protection policies, resulting in weaker regulations and potentially less severe penalties for such enterprises.

Additionally, differences in the production processes and emission levels of enterprises across industries contribute to the varying impacts of policies. In heavily polluting industries such as iron and steel and chemical industries, production processes and emission levels are typically complex, making it challenging for these enterprises to fully comply with environmental regulations and reduce pollution emissions. Conversely, in lightly polluting industries like electronic equipment, textile, and equipment manufacturing, production processes, and emission levels are relatively straightforward, making it easier for these enterprises to adhere to environmental regulations. These differences in production processes and emission levels across industries also contribute to the variations in the impact of RCS on pollution levels among enterprises.

## 5 Mechanism analysis and testing

### 5.1 Mechanism analysis

Compared with the traditional administrative unit to control pollution, the influence mechanism of RCS is mainly embodied in the “two hands” of the government and the market, to reduce the pollution emissions of enterprises and promote the improvement of environmental quality.

Specifically, under the traditional administrative model, water environment management falls under the responsibility of environmental protection departments at various government levels, typically relying on administrative orders and regulatory tools. In contrast, RCS introduces a more flexible and collaborative management system through the establishment of a flattened management approach. This is achieved by implementing a RCS, which reduces the number of management tiers and clarifies the responsibilities and division of labor for each individual in charge of managing a specific river. Moreover, RCS promotes a pattern of multiple participation and collaborative governance involving the government, social organizations, and the public. This inclusive approach enables diverse stakeholders to contribute to river governance and strengthens the clarity of governance responsibilities and the transparency of monitoring mechanisms. By adopting this flattened model, the effectiveness of water environment governance is enhanced, leading to improvements in the sustainable use and protection of water resources.

Throughout the different stages of policy implementation, including the initial phase, the supervisory stage, and subsequent rewards and punishments, the government’s coercive power plays a pivotal role in driving policy improvement and development. To ensure the quality and effectiveness of emission control efforts, the government needs to establish robust and comprehensive rules and regulations, continually adjust and enhance measures aimed at reducing pollutant emissions, and facilitate the smooth implementation of the policy.

The implementation of RCS not only raises the environmental awareness of regional enterprises and underscores the significance of environmental protection in their production and manufacturing processes but also serves as a testing ground for the government to experiment with new forms of environmental governance, ultimately improving the ecological environment’s quality within the region. Additionally, RCS enhances the implementation process of enterprise pollution control, improves the business environment to a certain extent, promotes the maturation of market mechanisms, and grants enterprises greater autonomy in decision-making and self-regulation. Consequently, RCS can attract more enterprises to relocate to the region and contribute to the enhancement of regional economic development. Furthermore, as a result of heightened environmental protection requirements for enterprises following RCS implementation and the imposition of stricter penalties for environmental pollution, enterprises will be prompted to adopt more environmentally friendly and low-carbon production methods and technologies. This, in turn, promotes the transformation and upgrading of enterprises, enhancing their technological content and competitiveness.

**Figure 3:**
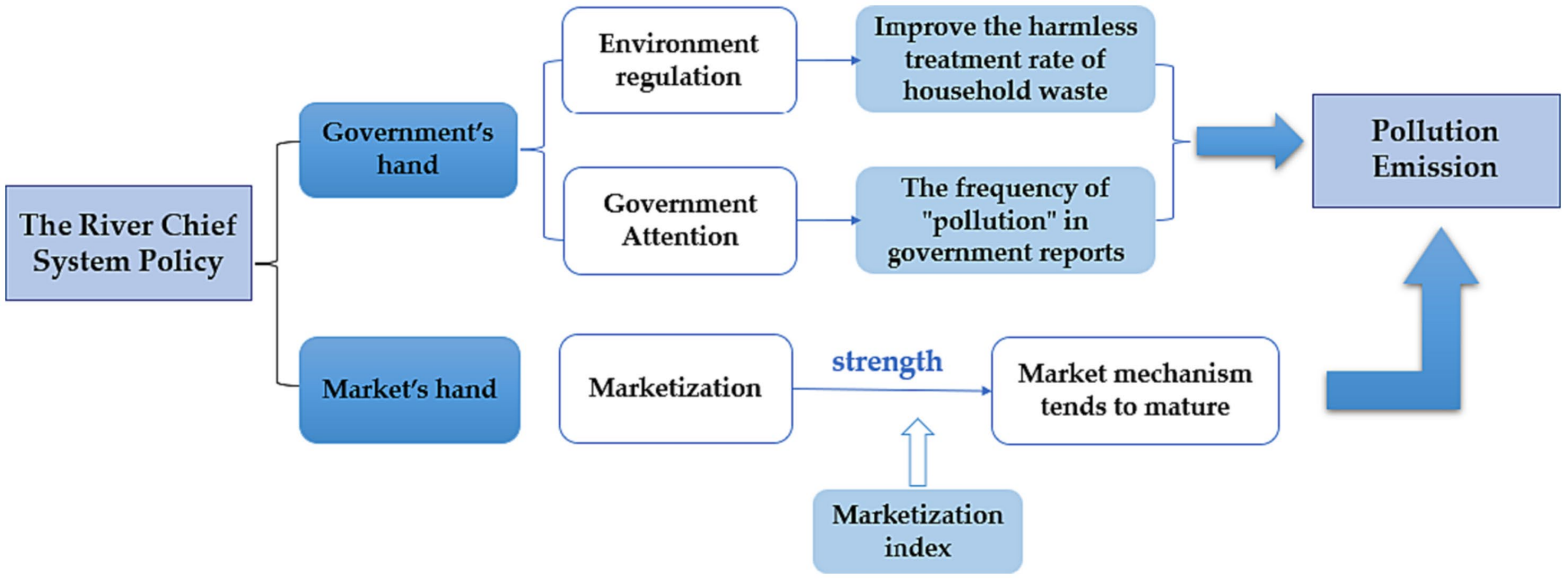
The mechanism of RCS influence on pollution emission of enterprises.

To provide evidence that the implementation of RCS effectively reduces the extent of pollution emissions from local enterprises and contributes to the improvement of the regional ecological environment, this study employs RCS as a policy shock and conducts a specific mechanism test. The analytical results and findings regarding this mechanism can be referred to in Figure 3, which illustrates the impact of RCS on enterprise pollution emissions.

### 5.2 Mechanism testing

#### 5.2.1 “The hand of government”

The introduction of RCS has brought about positive changes in river management (32). In contrast to the previous approach of dividing administrative boundaries, RCS emphasizes information sharing, openness, and transparency through the establishment of a flattened management structure. This allows relevant information to be more widely accessible and monitored by the public, resulting in more professional and efficient management of specific rivers. The implementation of RCS strengthens the involvement of government departments at various levels, from higher authorities to lower levels, throughout the policy implementation process (32). River managers, who may be local government officials or heads of relevant departments, hold significant decision-making and operational powers (58). They are better positioned to coordinate cooperation between departments and enhance the overall effectiveness of pollution control measures (34). Thus, the “government’s hand” is crucial in the implementation of RCS.

The level of government attention to environmental protection is closely intertwined with the implementation of RCS (34). In this study, the frequency of the term “pollution” in the government work reports of each prefecture-level city from 2003 to 2013 was manually collected as an indicator of the government’s focus on environmental protection. The results demonstrate that there is a strong correlation between the government’s concern for environmental pollution and the implementation of RCS, as evidenced by the higher frequency of the term “pollution” being mentioned in the reports when the government pays greater attention to environmental protection. Further analysis utilizing this indicator as a semi-mediated variable is presented in Table 10, revealing that the government’s concern for environmental pollution increases alongside the implementation of RCS. The more emphasis the government places on environmental protection and pollution control, the more frequently the term “pollution” is mentioned in its annual reports. This reflects the government’s recognition of environmental pollution as a significant issue and underscores its importance and progress in addressing it through the annual reports. Furthermore, when the government includes the term “pollution” in its annual reports, it may serve to highlight the government’s efforts, significance, and progress in addressing pollution in specific rivers. This underscores the government’s acknowledgment of environmental pollution as a pressing challenge and emphasizes the progress and importance of addressing it in the annual reports. As the government pays more attention to environmental protection, its regulation of enterprises’ environmental behavior strengthens, subsequently leading to more proactive environmental protection measures and a further reduction in pollution emissions levels (59). Therefore, it can be concluded that the level of government attention to environmental protection and the implementation of RCS jointly promote environmental protection actions by enterprises, ultimately achieving pollution management and reduction. This indicates that the government plays a crucial role in the implementation of RCS by focusing on and regulating environmental issues, thereby promoting enterprises’ environmental behavior and pollution control measures.

**Table 10:** Mechanism testing of government attention to environmental protection.

	Fixed effects of panel data
	Frequency of the word “pollution”
	*lox_w*
*Policy*	0.074***
	(2.77)
Year fixed effect	Yes
Firm fixed effect	Yes
Control Variables	Yes
Observations	196,776
Adj. R-squared	0.266

To investigate the impact of environmental regulations on enterprise pollution discharge, this study utilizes the centralized treatment rate of sewage treatment plants in prefecture-level cities as a proxy variable for environmental regulation. This indicator reflects the level of investment and management efforts by local governments in sewage treatment and water quality regulation (60). Based on the data presented in Table 8, it is evident that local governments have increased their investment in environmental regulation, thereby enhancing the regulation and management of sewage treatment. This has effectively reduced the negative impact of sewage discharge on the environment, thereby safeguarding public health and the quality of the living environment.

The implementation of the RCS has reinforced the responsibility and supervision of local governments in water resource management, consequently promoting an increase in the centralized treatment rate of wastewater treatment plants. This system also provides stricter management and supervision of water pollution discharges from enterprises (34). Hence, the rise in the centralized treatment rate of wastewater treatment plants in prefecture-level cities can indicate the success of the river management system’s implementation. Additionally, it may also reflect an increased enthusiasm among local enterprises in managing pollution, with enterprises placing more emphasis on environmental protection and reducing pollution discharge (61). These findings suggest that the RCS, as a form of environmental regulation, has a positive impact on enhancing the environmental awareness of enterprises and reducing pollution discharge by strengthening regulation and management of wastewater treatment (Table 11).

**Table 11:** Mechanism testing of environment regulations.

	Fixed effects of panel data
	Environmental regulations
	*lox_w*
*Policy*	0.022***
	(5.98)
Year fixed effect	Yes
Firm fixed effect	Yes
Control Variables	Yes
Observations	119,773
Adj. R-squared	0.757

#### 5.2.2 “The hand of the market”

However, in the implementation of RCS, relying solely on government efforts is insufficient, as it requires the active participation of multiple stakeholders, including the market. The market serves as a meeting point for all stakeholders, prompting the government, enterprises, and the public to pay attention to and participate in environmental protection (62). Through the operation of market mechanisms, the effective allocation of resources, the development of environmentally friendly technologies, and sustainable economic development can be promoted (41). Market participation also facilitates information circulation, information sharing, and the formation of a shared sense of responsibility and action (56). When the government, enterprises, and the public participate collectively in the market, they synergize and jointly promote environmental protection and the achievement of sustainable development goals.

The role of the regional enterprise business environment in the implementation of RCS is highly significant and worth investigating. In this study, the marketization index of prefecture-level cities from 2003 to 2013 is adopted as a measure of the regional enterprise business environment. An interaction analysis is conducted between this index and RCS, with the specific results presented in Table 8. It can be concluded from Table 8 that regions with higher marketization indexes have more mature enterprise business environments, allowing enterprises to possess greater decision-making and self-binding powers. Simultaneously, the implementation of RCS encourages enterprises to reduce emissions. The improved business environment provides enterprises with increased motivation and freedom to take proactive measures. Therefore, as the business environment matures, enterprises are more inclined to implement emission reduction measures and promote environmental protection, ultimately reducing pollution emissions levels within the region. It can be said that RCS and the business environment (marketization index) interact with each other, synergistically promoting the environmental protection actions of enterprises and collectively achieving pollution treatment and reduction (Table 12).

**Table 12:** Mechanism testing of enterprise business environment.

	Fixed effects of panel data
	Market index
	*lox_w*
*Index*	0.000
	(.)
*c.policy#c.index*	−0.054***
	(−3.03)
Year fixed effect	Yes
Firm fixed effect	Yes
Control Variables	Yes
Observations	257,382
Adj. R-squared	0.776

Based on the mechanism tested in this study, it can be concluded that the implementation of RCS has increased the government’s focus on environmental protection and strengthened its regulatory efforts. These measures have effectively reduced the level of pollution emissions from enterprises, significantly improving the environmental quality of the region and providing favorable conditions for enterprise transformation and modernization. Thus, the implementation of RCS has played a vital role in promoting environmental protection while also yielding economic and social benefits. It is anticipated that in future environmental protection endeavors, both the government and enterprises will give greater attention to environmental preservation and sustainable development. They will actively promote the implementation of RCS, fostering a virtuous cycle of environmental protection and economic development.

## 6 Conclusions and discussions

### 6.1 Marginal contributions and limitations

In terms of research perspectives, the RCS, in contrast to the traditional way of managing pollution through administrative units, embraces the principle of flat management to enhance the management and protection of water resources. This policy has yielded positive outcomes in environmental governance, concurrently mitigating bureaucratic hurdles and bolstering management efficiency. It has ushered in innovations across management systems, the delineation of responsibilities, oversight mechanisms, long-term sustainability, and collaborative governance. Environmental sustainability is crucial for human well-being, and RCS, as an innovative pollution control mechanism, plays a vital role in improving river water quality and reducing corporate pollution discharge, thereby mitigating potential threats to public health. Therefore, our study provides the government with new perspectives on RCS and valuable references for its successful implementation, thereby promoting environmental sustainability and safeguarding public health. Regarding the study sample, we pay special attention to the effects of RCS at the prefecture-level city level, which sets it apart from previous studies that mainly focused on the provincial and national levels. By surveying and studying enterprises at the prefectural and municipal levels, we gain a more accurate understanding of the policy’s impact at the local implementation level and conduct a more nuanced examination of the challenges faced by public health in different regions. This enables local governments to provide more precise solutions to local problems and ensure the health and well-being of the local public. In terms of research strategy, we delve into the key mechanisms affecting the level of pollution emissions from enterprises, providing local governments with more specific and actionable policy recommendations to promote environmental improvement and the sustainable development of enterprises. These policy recommendations include strengthening cooperation and communication with enterprises, enhancing their environmental awareness and actions, further reducing pollution emissions, improving environmental quality, and adopting targeted public health protection measures. These measures will enhance the efficiency and quality of local governance and provide robust protection for public health and environmental preservation.

All in all, the study serves as an important reference for the government’s decision-making process, as it not only promotes the improvement of environmental quality but also contributes positively to public health. By promoting the implementation of RCS, the government can better realize the Sustainable Development Goals (SDGs), safeguard public health and make lasting contributions to human well-being.

However, there are several limitations to acknowledge in this study. Firstly, the period of the study from 2003 to 2013 may not capture recent developments due to the constraints of policy timeframes. Nonetheless, our findings reveal a significant negative correlation between RCS and the extent of pollution discharged by enterprises, and we believe that this effect still holds. Secondly, the measurement of the extent of pollution discharge was based on river COD. While COD provides some insights into the level of pollution discharge by enterprises, it is not a comprehensive indicator. We acknowledge that incorporating additional indicators may offer a more comprehensive understanding of pollution discharge, and future studies could explore the inclusion of other relevant indicators. Despite these limitations, this study offers important insights into the impact of RCS on enterprise river pollution and serves as a foundation for further exploration and enhancement of river management policies.

### 6.2 Recommendations

On this basis, we present the following recommendations for the future implementation of RCS.

Firstly, RCS represents a crucial and innovative environmental policy adopted by local governments in China. Our study reveals that RCS can effectively reduce pollution emissions from firms, thus facilitating the enhancement of China’s environmental protection management system. Moreover, apart from its impact on environmental protection, RCS also plays a pivotal role in public health. By curbing the emission of pollutants, especially harmful substances in the air and water, RCS contributes to the amelioration of people’s living environment and health. The improvement in air and water quality decreases people’s exposure to hazardous pollutants, consequently reducing the risks of respiratory diseases, cardiovascular issues, and other ailments associated with pollutant exposure. Local governments should intensify their efforts in implementing RCS, ensuring effective execution of the policy while giving due consideration to public health concerns during the implementation process. Additionally, the government should provide comprehensive training and guidance to river chiefs to enhance their expertise in river management and environmental protection, thereby safeguarding river ecosystems and ensuring public drinking water safety and health.

Furthermore, research indicates that by increasing the government’s attention to environmental protection and bolstering environmental regulations, RCS has effectively reduced pollution emissions from enterprises. Consequently, local governments should elevate their focus on environmental pollution issues and prioritize public health in environmental protection initiatives. By disseminating more detailed and transparent environmental reports, establishing an environmental information disclosure platform, and regularly publishing river water quality monitoring results and corporate discharge information, public awareness and attention to river pollution problems can be raised. Simultaneously, encouraging public participation in environmental protection inspections and monitoring will strengthen social supervision, ensuring that policies align with public health interests. Establishing effective communication channels between the government, enterprises, and the public is essential, considering public opinions and needs to foster a collaborative approach toward environmental protection and public health. Encouraging active involvement from the general public, non-governmental organizations, and businesses in the management and protection of river waters will create a participatory and synergistic governance framework, leading to improved ecological environments and enhanced public health. Additionally, the government should intensify its supervision and enforcement of enterprises, particularly those engaging in activities that impact public health, ensuring that businesses’ operations do not compromise public health interests. Strengthening environmental protection regulations and standards and implementing stricter supervision and penalties for non-compliant enterprises will contribute to creating a healthier and safer living environment for the public.

Lastly, enhancing the business environment will further bolster the effectiveness of RCS in river pollution management, subsequently improving public health. Local governments are advised to implement market-oriented reforms, promoting competition among enterprises and fostering the efficient operation of market mechanisms. Encouraging the upgrading of industrial structures, reforming market access systems, and enhancing market supervision will elevate the overall business environment. Improved business conditions will not only incentivize enterprises to proactively engage in environmental protection but also enhance their sense of social responsibility and active participation in public health initiatives. This will motivate companies to prioritize pollution reduction and adopt more environmentally friendly production practices, thereby further enhancing public health.

In conclusion, by implementing the above recommendations, multi-party participation and cooperation between the government, enterprises, and the public can be strengthened, leading to the streamlined management and protection of rivers while considering public health considerations. This will drive the improvement and sustainable development of river ecosystems, ultimately creating a healthier and better living environment for the people.

## 7 Conclusion

This study utilizes a database of industrial enterprises from 2003 to 2013, along with manually organized RCS data for each prefecture-level city, which is used to conduct a difference-in-differences (DID) analysis to examine the impact of RCS on the degree of pollution emissions from enterprises. The findings of the study are as follows:

Firstly, RCS significantly reduces firms’ pollution emissions. This conclusion is corroborated by a series of robustness tests, including parallelism trend tests and placebo tests. Secondly, the impact of RCS on firms’ pollution emissions varies across different levels. The effectiveness of RCS in reducing observable pollutants is more pronounced when considering whether the pollutant is observable or not. This suggests that RCS may have a significant effect on reducing some observable pollutants that directly threaten public health. At the administrative level, the study found that RCS has a more substantial effect on lowering pollution emissions from firms located in non-provincial capitals. This indicates that RCS could be instrumental in improving local environmental quality and safeguarding public health in certain prefectural or county-level cities. Furthermore, concerning the degree of pollution, the research results demonstrate that RCS has a more prominent positive effect on improving river water quality for enterprises operating in heavily polluting industries. These industries often discharge large amounts of hazardous substances, posing a higher risk to the environment and public health. Consequently, the application of RCS in such industries may significantly impact public health. Thirdly, increasing government focus on environmental protection and strengthening environmental regulations are crucial avenues for RCS to reduce corporate pollution emissions. Proactive government involvement and effective environmental regulation can facilitate the successful implementation of RCS, further enhancing river water quality and safeguarding public health. Additionally, an improved business environment can augment the efficacy of RCS in reducing corporate pollutant discharges. Enterprises should actively adopt environmental protection measures to decrease pollutant emissions, thereby forming a robust synergy with RCS policies to further enhance environmental quality and public health.

In summary, the above findings unveil the significant impact of RCS on corporate pollution emissions and analyze its differential effects across various dimensions and factors. This provides a critical foundation for the government to formulate environmental policies and decisions, promoting the continued development and implementation of the RCS policy to preserve public health while enhancing river water quality.

## Data availability statement

The raw data supporting the conclusions of this article will be made available by the authors, without undue reservation.

## Author contributions

JD: Conceptualization, Data curation, Methodology, Writing – original draft. BL: Data curation, Supervision.
